# Redox Structures of Humic Acids Derived From Different Sediments and Their Effects on Microbial Reduction Reactions

**DOI:** 10.3389/fmicb.2018.01225

**Published:** 2018-06-08

**Authors:** Ning Zhang, Dong-Dong Zhang, Hong-Da Ji, Xin-Wei Yu, Zhi-Chao Zhang, Sheng-Mao Yang, Chun-Fang Zhang

**Affiliations:** ^1^Institute of Marine Biology, Ocean College, Zhejiang University, Hangzhou, China; ^2^Wuxi Dongfang Environmental Engineering Design and Research Institute, Wuxi, China; ^3^Zhoushan Municipal Center for Disease Control and Prevention, Zhoushan, China; ^4^Institute of Environment Resources and Soil Fertilizer, Zhejiang Academy of Agricultural Sciences, Hangzhou, China

**Keywords:** humic acid, electron mediator, elemental composition, spectroscopic analysis, nitrate reduction, FeOOH reduction, redox activity

## Abstract

Herein, we investigated the chemical, electrochemical, and spectroscopic characteristics of humic acids (HAs) extracted from sediments of different origin [Ling Qiao river, Xi Xi wetland, Qi Zhen lake (QZ), and Hu Zhou pond in Zhejiang province, China], paying particular attention to their role in the enhancement of nitrate and FeOOH reduction. Notably, the highest C/N ratio (16.16), O/C ratio (1.89), and Fe content (11.57 g kg^-1^ sample) were observed for HAs extracted from QZ sediment. Cyclic voltammetry analyses confirmed that all HAs contained redox-active groups and exhibited redox potentials between -0.36 and -0.28 V vs. the standard hydrogen electrode. All HAs showed similar Fourier transform infrared spectra with variable absorption intensity, the spectra verified the presence of aromatic C=C, C–H, and C=O of quinone ketones group in HAs. Electron spin resonance suggested that quinone moieties within HAs are the redox-active centers. All HAs promoted the microbial reduction of nitrate and amorphous FeOOH by *Shewanella oneidensis* strain MR-1, achieving high nitrate reduction extents of 79–98.4%, compared to the biotic and abiotic control values of 29.6 and 0.006%, respectively. The corresponding extents of Fe(II) production equaled 43.25–60.5%, exceeding those of biotic and abiotic controls (28.5 and 0.005%, respectively). In addition to the highest C/N, O/C ratio, and Fe content, HA extracted from QZ sediment also exhibited the highest nitrate and FeOOH reduction performances. Although the proportion of organic redox-active carbon is small, the potential electron-mediating ability is not ignorable. HAs are redox active for enhancing microbial reduction of nitrate and amorphous FeOOH regardless of the location or texture of parent sediments, implying their great potential for acting as redox mediator in enhancing multiple microbial reduction, thereby affecting various biogeochemical processes (i.e., iron cycle, nitrogen cycle, etc.) as well as *in situ* remediation in anaerobic environment.

## Introduction

Humic substances (HSs) are redox-active organic materials widely present in natural environment, e.g., in soils, sediments, peatlands, and wetlands (Lovley et al., 1996). According to their solubility, HSs can be divided into three groups (Aiken et al., 1985), namely humin, humic acids (HAs), and fulvic acids. Among them, HAs are a prominent fraction of HSs, significantly affecting the distribution, sorption, transport, and fate of environmental pollutants (Scott et al., 1998; Gu and Chen, 2003; Guo et al., 2018), e.g., the production and toxicity of trace metal contaminants, disinfection byproduct formation, adsorption and dissolution of hydrophobic organic pollutants, and redox reactions in soils.

Dissimilatory ${\mathrm{NO}}_{3}^{–}$ reduction is known to comprise two different processes, denoted as denitrification and ${\mathrm{NO}}_{3}^{–}$ reduction to ${\mathrm{NH}}_{4}^{+}$ (DNRA). The latter process, also known as ${\mathrm{NO}}_{3}^{–}$ ammonification or fermentative ${\mathrm{NO}}_{3}^{–}$ reduction, has recently attracted increased attention due to its significance for nitrogen cycling in various ecosystems, achieving greater N retention than denitrification, since the produced ${\mathrm{NH}}_{4}^{+}$ is more available for plant and microbial uptake but less prone to losses via leaching or volatilization compared with N_2_ produced by denitrification (Rütting et al., 2011). Dissimilatory microbial reduction of Fe(III) to Fe(II) plays a key role in environmental iron cycling and influences a number of biochemical processes such as the exchange of nutrients and trace metals between aquatic systems and sediments (Wang et al., 2017). Thus, both DNRA and FeOOH reduction significantly affect organic matter exchange and geochemical cycling in anoxic systems.

Reduction reactions under anaerobic conditions are commonly limited by the rate of electron transfer, which can be accelerated by the use of redox mediators (RMs), with certain reactions simply not taking place in their absence (Zhang and Katayama, 2012; Zhang et al., 2017). Over the past two decades, HSs and their quinoid model, anthraquinone-2,6-disulfonate (AQDS), have been reported to act as RMs for the reduction of several substrates (Gu and Chen, 2003; Costa et al., 2010; Zhang D.D. et al., 2015; Wang et al., 2017; Guo et al., 2018), e.g., chlorinated compounds, nitrate, azo dyes, and oxidized metal ions such as Fe(III), Cr(VI), and U(VI).

Humic acids have been hypothesized to contain redox-active functional groups such as quinones and have the ability to transfer electrons, accepting those generated by microbial lactate oxidation and donating them to nitrate or Fe(III)-containing minerals, thus accelerating the reduction of nitrate and FeOOH (Lovley et al., 1996; Wang et al., 2017). Although HAs (extracted from soils/surface waters) and their quinoid analogs (e.g., AQDS) have been extensively characterized, the redox characterization of HAs extracted from different sediments and their functions as RMs for microbial nitrate and Fe reduction has been underexplored. Moreover, owing to the complexity and heterogeneity of HAs, their structures are difficult to determine by conventional methods. Therefore, to better understand the reactivity of HAs originating from different sediments, these species were herein characterized by chemical, electro(chemical), and spectroscopic methods, and their effects on nitrate and FeOOH reduction by model dissimilatory metal-reducing bacteria (*Shewanella oneidensis* MR-1) were also determined, shedding light on the underlying reasons of different electron-mediating efficiencies of HAs on microbial metabolic processes.

## Materials and Methods

### Sediment Samples

Sediment samples were obtained from four different locations [Ling Qiao river (LQ) (29° 51′ 59″ N, 121° 33′ 35″ E), Xi Xi wetland (XX) (30° 15′ 27″ N, 120° 3′ 25″ E), Qi Zhen lake (QZ) (30° 18′ 28″ N, 120° 5′ 3″ E), and Hu Zhou pond (HZ) (30° 46′ 38″ N, 120° 9′ 5″ E)] in Zhejiang province, China. LQ represents an area with pollution, there are many factories around it, such as detergent factories, paper mills, etc. XX is inside the nature reserve area with a lot of vegetation (such as herbs and shrubs). QZ represents an area with low pollution, which is located in Zijingang campus of Zhejiang University. HZ sediment was sampled from aquaculture pond cultured with black carp. The collected samples were air-dried and stored at room temperature prior to use. Further description of physicochemical characteristics of the sampling sites and interstitial water in the sediments is shown in Supplementary Table S1 of Supplementary Material.

### Extraction and Purification of HAs

Humic acids were extracted from air-dried sediment samples as described previously (Zhang and Katayama, 2012). Briefly, 500 g of sediments treated with 2 vol% HF (Schmidt et al., 1997) was shaken with 0.1 M NaOH at 150-170 rpm for 24 h and centrifuged, with the supernatant collected as the extract. The above process was repeated at least eight times until the dark color of the extract was not obvious. The collected alkaline extract was acidified to pH < 2 with 2 M HCl and coagulated in the dark overnight, with the produced precipitate (HAs) subsequently collected by centrifugation and subjected to repeated alkali and acid treatment to remove the residual inorganic particulate matter. Finally, the obtained HAs were rinsed with HF once and then repeatedly rinsed with distilled water and freeze-dried. All the solutions and distilled water used during the extraction were flushed with N_2_, and all the centrifugation steps were carried out at 5000 × *g* for 10 min. Samples extracted from sediment collected from the LQ, XX, QZ, and HZ were denoted as LQ-HA, XX-HA, QZ-HA, and HZ-HA, respectively.

Four HAs stock solutions were separately prepared by dissolving HAs in deionized water containing 2 ml of 0.1 M NaOH solution under nitrogen, with the pH subsequently adjusted to 7.2 with 1 M HCl. A standard commercial (Aldrich) HA stock solution was also prepared in the same way. Thus the obtained stock solution was deoxygenated by purging with N_2_ and used immediately (Zhang and Katayama, 2012).

### (Electro)chemical Analyses

The C, H, N, and S contents of HAs were determined using a Yanaco MT-5 CHN-corder (Yanaco New Science Inc., Kyoto, Japan) with a high-temperature combustion method. Ash contents were determined by combustion of dried HAs at 600°C for 5 h. Oxygen contents were calculated by the mass difference. Metal contents were determined by inductively coupled plasma atomic emission spectroscopy (Optima 3300DV, PerkinElmer, Yokohama, Japan) after perchloric acid and nitric acid digestion, the procedure of acid digestion is described as follows: 0.3 g of each HA sample was accurately weighted and placed at 50 ml Erlenmeyer flask, then digested with about 8.0 ml of mixed acid solution (the volume ratio of nitric acid to perchloric acid was 4:1) on an electrothermal board at 120°C to keep micro-boiling state. When the brown smoke disappeared and the sample was nearly transparent, continue heating to near dryness, cooling, finally diluted to 20 ml with 2% nitric acid.

Cyclic voltammetry (CV) measurements were performed using a potentiostat (HSV-110; Hokuto Denko Inc., Osaka, Japan) comprising twisted Pt (0.8 mm × 1 m, Nilaco, Tokyo, Japan) as working and counter electrodes and a Ag/AgCl reference electrode (6 mm × 15 cm, Fusheng Analytical Instrument Co., Shanghai, China). To perform these measurements, HA samples (0.25 g) were suspended in 1 mM NaClO_4_ in dimethyl sulfoxide (DMSO, 50 ml) as an electrolyte (Nurmi and Tratnyek, 2002), and CV curves were recorded at a scan rate of 10 mV s^-1^ within a potential range of -2.0 to 0.0 V vs. Ag/AgCl. All test solutions were purged with high-purity N_2_ for 15 min before each set of scans, and background scans were performed before every measurement.

Fourier transform infrared (FTIR) spectra of HAs were recorded on a JASCO FT/IR-6 100 spectrometer (JASCO, Tokyo, Japan) in the range of 400–4000 cm^-1^ at a resolution of 4 cm^-1^ using eight scans. The KBr pellet technique was applied, and the recorded spectra were background-corrected using pure KBr and ambient air as blanks (Francioso et al., 1998).

Electron spin resonance (ESR) was used to measure the organic radical contents of HSs (Roden et al., 2010). The signal intensity of the ESR radicals of each HA was examined. The pH of HA was adjusted using either 0.1 M HCl or 0.1 M NaOH to pH 3 or 11, and freeze-dried. The ESR spectra were recorded at room temperature using a Bruker ESRA-300 spectrometer (Berlin-Adlershof, Germany), operating at 9.85 GHz with a 100 kHz modulation frequency of the steady magnetic field. The freeze-dried HA samples were measured in quartz glass sample tubes with an inner diameter of 1 mm. Other spectrometric conditions for the measurements were as follows: magnetic field centered at 3500 G, 6.37 mW microwave power, 20.5 s sweep time, and 0.08 s time constant.

### Bacterial Culturing and Growth Conditions

The model dissimilatory metal-reducing bacteria (*S. oneidensis* strain MR-1) purchased from China Center for Type Culture Collection was used for microbial reduction experiments. The above bacteria were aerobically inoculated in a Luria-Bertani medium (10 g l^-1^ NaCl, 5 g l^-1^ yeast extract, 10 g l^-1^ tryptone) for 12 h in a shaker at 150-170 rpm, 30°C, being subsequently harvested by 3 × 10-min centrifugation at 8000 ×*g* and 4°C in phosphate buffer shortly before reaching the exponential growth phase. Washed late-log-phase cultures (2 × 10^7^ cells ml^-1^) of MR-1 were incubated in anaerobic HA medium, which was prepared in 50-ml bottles containing 20 ml of mineral medium (Zhang et al., 2014), 0.2-μm-filter-sterilized vitamin solution, 125 mg l^-1^ HA solution, and 10 mM formate. The mineral medium comprised (per liter) 1.0 g of NH_4_Cl, 0.05 g of CaCl_2_⋅2H_2_O, 0.1 g of MgCl_2_⋅6H_2_O, 0.4 g of K_2_HPO_4_, 1 ml of trace element SL-10 solution, 1 ml of Se/W solution, 15 mM 3-(*N*-morpholino)propanesulfonic acid (MOPS) buffer (pH 7.2), and 50 mg resazurin-Na.

Nitrate (5 mM) or amorphous FeOOH (4 mM) was added as electron acceptors, and the bottles were sealed with Teflon-coated butyl rubber stoppers and aluminum seals and autoclaved at 121°C for 30 min. The cultures were statically incubated in the dark at 30°C. A 4-mM solution of amorphous FeOOH was prepared as described elsewhere (Lovley et al., 1996). Herein, the nitrate- and iron-reducing cultures are referred to as ${\mathrm{NO}}_{3}^{–}$-HA and Fe-HA cultures, respectively. For all conditions, triplicate cultures were prepared in addition to an autoclaved control (abiotic control) and an HA-free control (biotic control), and each culture was measured three times.

In the above cultures, ${\mathrm{NO}}_{3}^{–}$ was quantified by ultraviolet spectrophotometry (Cawse, 1967), and ${\mathrm{NH}}_{4}^{+}$ was quantified by Nessler’s reagent colorimetric method (Lee and Takahashi, 1966). The concentration of Fe(II) was measured spectrophotometrically using 1,10-phenanthroline (Jones et al., 1981).

## Results

### Chemical Characterization

The yield (by weight), nonmetalloid (C, H, N, S, O) element contents and ash contents of HAs are shown in **Table 1**. Specifically, the above yields varied from 1.64 to 15.04%, whereas the elemental compositions (containing ash) varied in the range of 8.26–15.27% for C, 1.30–2.89% for H, 0.65–1.21% for N, 0.35–0.93% for S, and 12.62–25.41% for O, the ash contents of HAs varied from 55.1 to 76.2%. C/N, H/C, and O/C atomic ratios were calculated as 12.88–16.16, 1.89–3.45, and 1.15–1.89, respectively, differing widely for different HAs.

**Table 1:** The physicochemical properties of HA samples.

HAs^a^	Yield (%)	Elemental composition (%)						C/N	H/C	O/C
		C	H	N	S	O	Ash (%)			
LQ-HA	1.64	8.26 ± 0.32	1.30 ± 0.05	0.65 ± 0.03	0.93 ± 0.03	12.62 ± 0.62	76.2	14.83 ± 0.18	1.89 ± 0.01	1.15 ± 0.48
XX-HA	10.06	10.05 ± 0.21	2.89 ± 0.04	0.91 ± 0.04	0.62 ± 0.07	16.60 ± 0.20	68.9	12.88 ± 0.92	3.45 ± 0.11	1.24 ± 0.08
QZ-HA	15.04	9.69 ± 0.34	1.82 ± 0.23	0.70 ± 0.03	0.35 ± 0.01	24.44 ± 0.59	63.0	16.16 ± 1.05	2.25 ± 0.02	1.89 ± 0.28
HZ-HA	5.75	15.27 ± 0.34	2.54 ± 0.08	1.21 ± 0.02	0.45 ± 0.02	25.41 ± 0.39	55.1	14.72 ± 0.55	2.00 ± 0.06	1.25 ± 0.08

*^*a*^HAs were extracted from sediment collected in Ling Qiao river (LQ-HA), Xi Xi wetland (XX-HA), Qi Zhen lake (QZ-HA), Hu Zhou pond (HZ-HA). Data show the mean values of four data sets.*

**Table 2** shows the metal (Fe, Cu, Mn, Zn, Cd, Cr, Pb) element contents of HAs. All HAs contained large amounts of Fe (9.82–11.57 mg per g HA) and Cu (0.58–1.46 mg per g HA), while the concentrations of Mn, Zn, and Cr equaled 0.07–0.14, 0.07–0.18, and 0.09–0.11 mg per g HA, respectively. The concentration of Cd was below the detection limit, except for QZ-HA (0.001 mg per g HA), similarly to that of Pb, except for LQ-HA (0.003 mg per g HA).

**Table 2:** Concentrations of metals in HA samples (mg per g HA).

HAs	Fe	Cu	Mn	Zn	Cr	Cd	Pb
LQ-HA	9.82 ± 0.02	1.46 ± 0.002	0.07 ± 0.001	0.07 ± 0.001	0.11 ± 0.001	ND	0.003 ± 0.0002
XX-HA	11.16 ± 0.02	0.58 ± 0.001	0.11 ± 0.001	0.07 ± 0.002	0.10 ± 0.001	ND	ND
QZ-HA	11.57 ± 0.01	0.74 ± 0.001	0.14 ± 0.001	0.08 ± 0.002	0.11 ± 0.001	0.001 ± 0.0001	ND
HZ-HA	11.21 ± 0.02	0.83 ± 0.001	0.12 ± 0.001	0.18 ± 0.001	0.09 ± 0.001	ND	ND

*ND denotes below the detection limit. Data show the mean values of three data sets. Abbreviated names of HAs are the same as those presented in **Table 1**.*

### Electrochemical Characterization

To assess the redox-active moieties of HAs, CV experiments were carried out using DMSO as a solvent to increase the electrode reactivity of HAs, since HSs generally produce CV curves with little or no useful structure due to lack of electrode activity (Sawyer et al., 1995).

**FIGURE 1:**
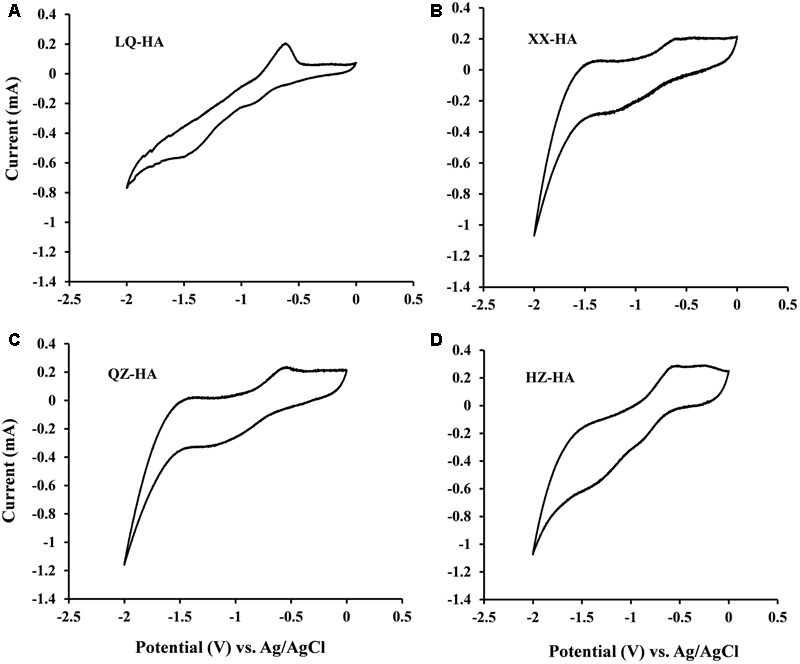
Cyclic voltammograms of different HAs [LQ-HA **(A)**, XX-HA **(B)**, QZ-HA **(C)**, HZ-HA **(D)**] dissolved in DMSO. Abbreviated names of HAs are the same as those presented in **Table 1**.

Redox couples were observed for all HA samples (**Figure 1**), and the corresponding redox potentials were estimated from CV curves recorded in DMSO. The potentials obtained in DMSO (vs. Ag/AgCl) were approximately 0.45 V higher than those in H_2_O [vs. the standard hydrogen electrode (SHE)] (Nurmi and Tratnyek, 2002), with the validity of this correction confirmed by our previous research on humins (Zhang and Katayama, 2012). The redox potentials of LQ-HA, XX-HA, QZ-HA, and HZ-HA were estimated as -0.3, -0.32, -0.28, and -0.36 V (vs. SHE), respectively.

### Spectroscopic Characterization

**FIGURE 2:**
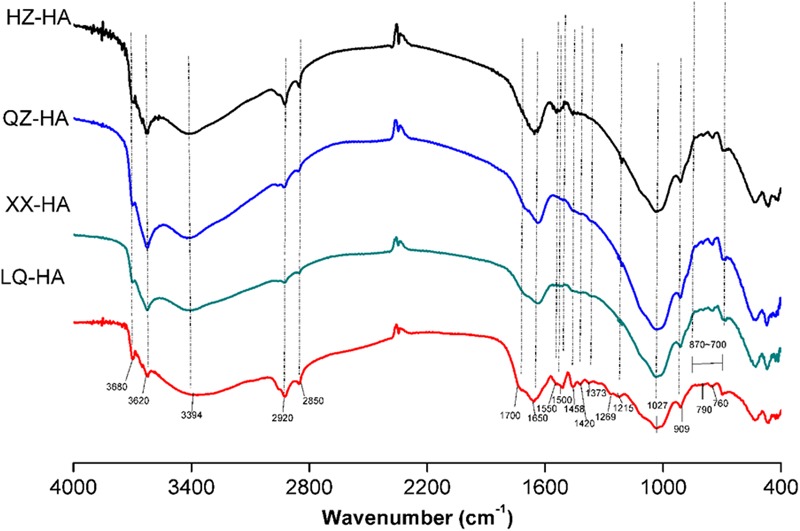
Fourier transform infrared (FTIR) spectra of HAs extracted from four different sediments. Abbreviated names of HAs are the same as those presented in **Table 1**.

Fourier transform infrared spectra are shown in **Figure 2**. Interpretation of the spectra were based on those literatures (Francioso et al., 1998; Senesi et al., 2003; Hesse et al., 2005; Tatzber et al., 2007).

All HAs showed bands around 3680 and 3620 cm^-1^ as well as a broad peak around 3394 cm^-1^, attributed to O–H vibrations of carboxylic or phenolic groups and to N–H stretches of amides and amines. Peaks around 2920 and 2850 cm^-1^ were assigned to symmetric and asymmetric vibrations of aliphatic CH_3_ and CH_2_ groups. A small peak at 1700 cm^-1^ could be attributed to carbonyl vibrations of carboxyl groups, esters, aldehydes, and ketones. A pronounced peak at 1650 cm^-1^ was ascribed to C=C stretches of aromatic rings, C=O stretches of amide groups (amide I band), and C=O of quinone ketones, and asymmetrical C–O stretches of carboxyl groups, which are commonly observed for HAs (Zhang C. et al., 2015).

Ling Qiao river-HA also showed a high resolved fingerprint area (from 1600 to 900 cm^-1^), whereas these spectra were not obvious in other HAs probably because of the low proportion of the corresponding groups. As for these spectra, the peak at 1550 cm^-1^ was assigned to COO–, the peak at 1500 cm^-1^ was assigned to the N–H, C=N (amide II band), and C=C, the peak at 1458 cm^-1^ was assigned to aliphatic C–H, the peak at 1420 cm^-1^ could be attributed to C=N of primary amides (amide III band), the peak at 1373 cm^-1^ could be attributed to –CO–CH_3_ and possibly from nitrate, the peak at 1269 cm^-1^ could be attributed to C–O stretching of aryl ethers, the peak at 1215 cm^-1^ could be attributed to C–O and OH of COOH, C–O of aryl ethers and phenols, P–O–aryl and tertiary alcohols, and a strong band at 1027 cm^-1^ could be attributed to C–O stretching of alcohol, sulfoxides, carbohydrates, or polysaccharides-like substances or Si–O of silicates. The band at 909 cm^-1^ was assigned to an aromatic C–H deformation. Meanwhile, the bands from 870 to 700 cm^-1^ could be attributed to aromatic C–H, and less substituted rings appear at less wave numbers, the peak at 790 cm^-1^ could be attributed to benzene rings with two or three adjacent H and/or an isolated H, R_2_C=CRH groups and nitrates, the peak at 760 cm^-1^ could be attributed to sp^3^–CH_2_ and benzene rings with four and/or five adjacent H.

**FIGURE 3:**
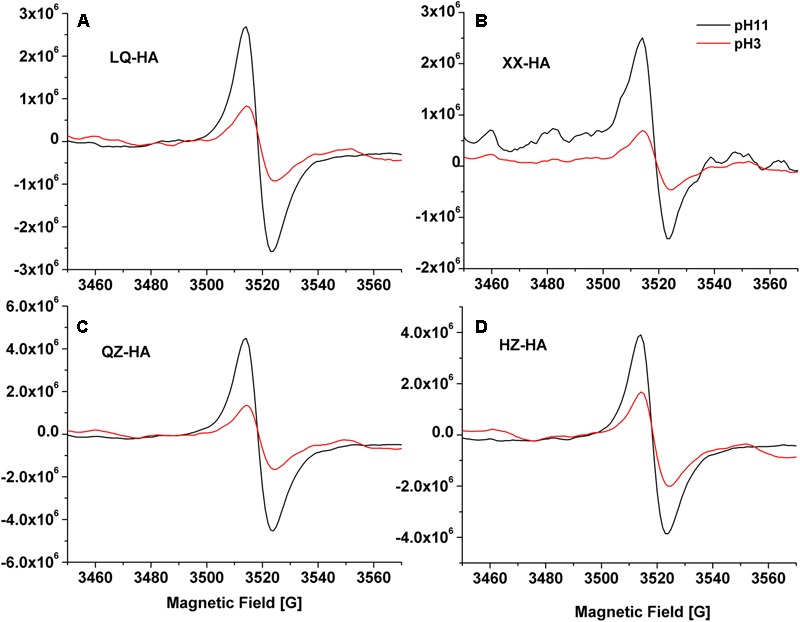
Electron Spin Resonance (ESR) spectra of different HAs [LQ-HA **(A)**, XX-HA **(B)**, QZ-HA **(C)**, HZ-HA **(D)**] samples. Abbreviated names of HAs are the same as those presented in **Table 1**.

Electron spin resonance spectra of HAs are shown in **Figure 3**. The results showed that all of the spectra for HAs were devoid of hyperfine splitting. The spectroscopic splitting constants (g) were in the range of 2.0041–2.0046, HZ-HA showed an ESR signal at *g* = 2.0046, While XX-HA at 2.0044, QZ-HA at 2.0042, and LQ-HA at 2.0041, these values agrees with previously reported *g*-values for HSs with semi-quinones being the primary organic radicals (Scott et al., 1998). All HAs adjusted at pH 11 produced remarkable increase in radical signal compared with that of HAs at pH 3. The increase in ESR signal at high pH is typical for the semiquinone-type radicals, which supports the interpretation that quinone moieties were responsible for the observed signal increase (Paul et al., 2006; Zhang and Katayama, 2012). However, we cannot exclude the contribution of ESR signals from other origins such as thiols, nitrogen functional groups, or metal-organic complexes.

### HA-Promoted Microbial Reduction of Nitrate

**FIGURE 4:**
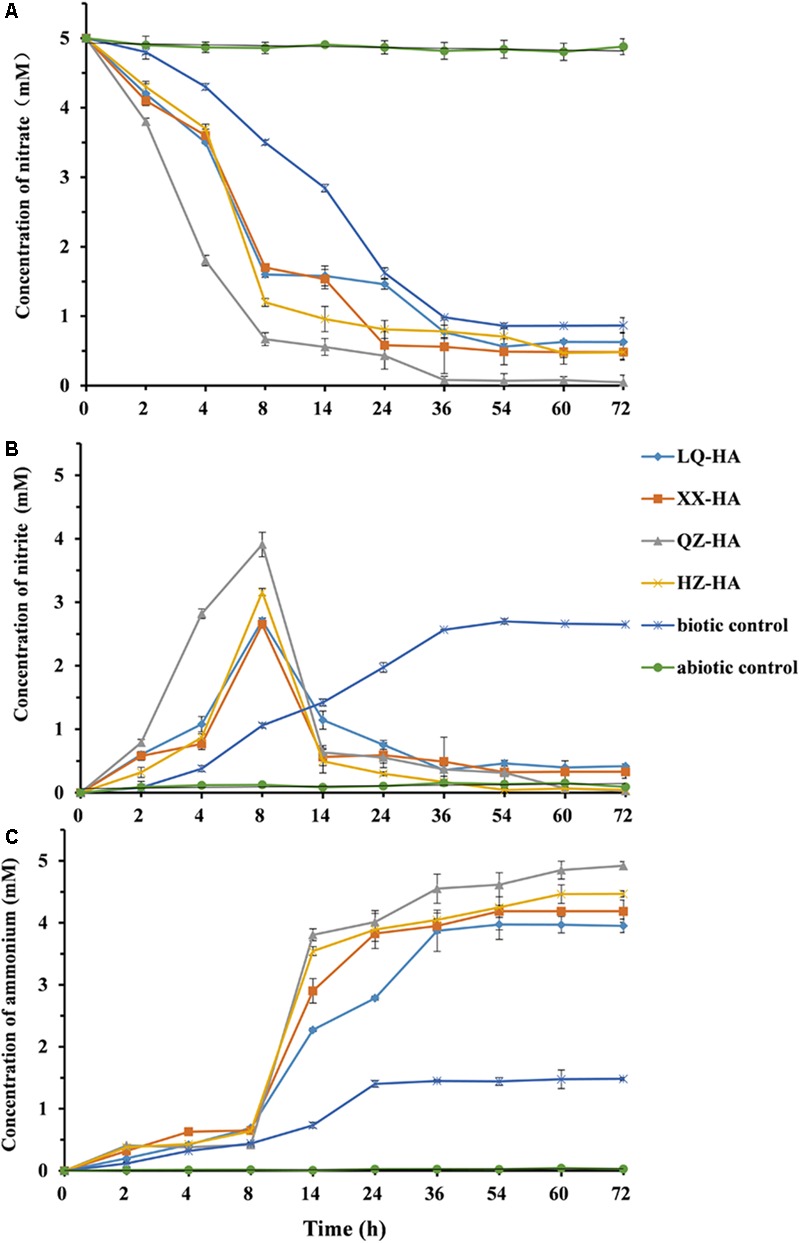
The changes in the concentration of nitrate **(A)**, nitrite **(B)**, ammonium **(C)** left in the culture within 72 h, in which microbial reduction were conducted by MR-1 in the presence of HAs of different origins (LQ-HA, XX-HA, QZ-HA, and HZ-HA) when 5 mM nitrate was added as the electron acceptor in each culture. Data show the mean values of triplicate cultures, and vertical bars show the difference in triplicate cultures.

**Figure 4** shows changes in the amounts of nitrate, nitrite, and ammonium observed during HA-promoted microbial reduction of 5 mM nitrate by MR-1. The biotic control corresponded to an HA-free culture, and the abiotic control was represented by an autoclave-sterilized culture. All HAs enhanced the microbial reduction of nitrate to different extents. Under nitrate-reducing conditions, the 5-mM nitrate was finally reduced to ammonium within 3 days (**Figures 4A,C**). The decrease of nitrate concentrations showed that the addition of HAs to the ${\mathrm{NO}}_{3}^{–}$-HA culture triggered anaerobic microbial DNRA without a lag phase. Compared to the biotic and abiotic controls, nitrate was initially reduced to nitrite, with the increase of ammonium concentration being slow within the first 8 h. Subsequently, the reduction of the produced nitrite to ammonium resulted in a rapid concentration increase of the latter between 8 and 14 h, with a plateau reached after 72 h. Among the four HAs, QZ-HA showed the best performance for nitrate reduction, achieving a reduction extent of 98.4% (4.92 mM ammonium in the culture), followed by HZ-HA (4.47 mM, 89.4%), XX-HA (4.19 mM, 83.8%), and LQ-HA (3.95 mM, 79%). However, for the biotic control, only ∼33% of nitrate could be transformed into ammonium, with the rest mainly converted to nitrite and accumulated, i.e., only 1.48 mM ammonium and ∼2.7 mM nitrite were produced. In the abiotic control sample, only 0.05 mM nitrite and 0.03 mM ammonium were observed, with the reduction extent thus equaling 0.006% (**Figure 4C**).

**Figures 4B,C** show that the nitrite concentration reached its maximum after 8 h, with the highest reaction extents observed for QZ-HA, and those of other cultures being similar to each other. In the time window of 8–72 h, ${\mathrm{NO}}_{3}^{–}$-HA cultures (except for the biotic control) mainly featured the reduction of nitrite to ammonium, with the highest reaction extent observed for QZ-HA, followed by HZ-HA, XX-HA, and LQ-HA in decreasing order. For the biotic control, the concentration of nitrite stabilized at 2.6 mM after 36 h, and that of ammonium stabilized at 1.45 mM after 24 h (**Figures 4B,C**). No nitrate consumption or nitrite and ammonium production was observed for all abiotic controls.

The observed changes in amounts of nitrate, nitrite, and ammonium as mediated by standard HA during microbial reduction of nitrate (5 mM) by MR-1 were showed in Supplementary Figure S1 of Supplementary Material.

### HA-Promoted Microbial Reduction of FeOOH

**FIGURE 5:**
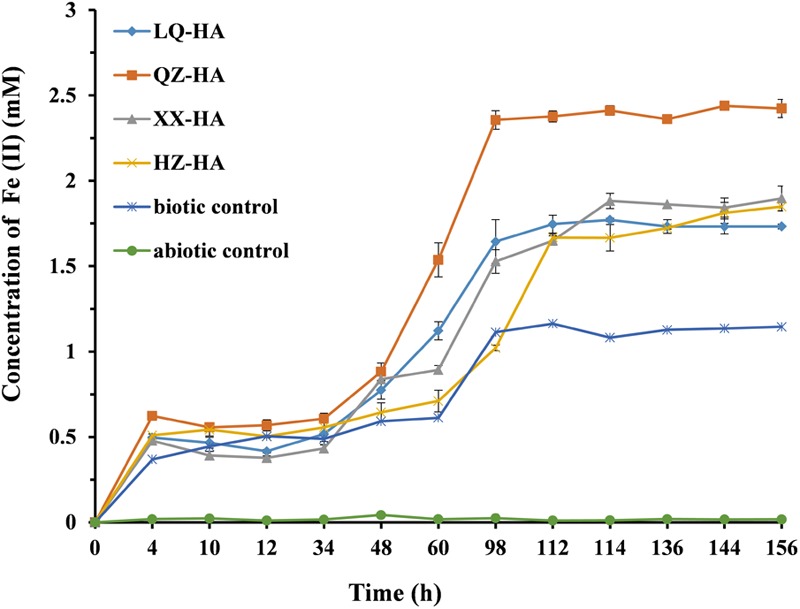
Production of total Fe(II) during the microbial reduction of FeOOH conducted by MR-1 in the presence of HAs of different origins (LQ-HA, XX-HA, QZ-HA, and HZ-HA) within 156 h. Data show the mean values of triplicate cultures, and vertical bars show the difference in triplicate cultures.

**Figure 5** shows the results of dissimilatory Fe(III) reduction by MR-1 in the presence of different HAs when 4 mM FeOOH was added as electron acceptor. In the Fe-HA culture, Fe(III) was reduced to Fe(II), with all HAs enhancing this process to variable extents within 156 h; as a result, 1.73–2.42 mM Fe(II) (reduction extent = 43.25–60.5%) was eventually produced in the presence of HAs. Conversely, in the HA-free biotic control, only 1.14 mM Fe(II) was detected, corresponding to a reduction extent of 28.5%, with the respective values for the abiotic control equaling 0.02 mM and 0.005%. The QZ-HA culture showed the best reduction performance, achieving a reduction extent of 60.5% [2.42 mM Fe(II)], followed by XX-HA (47.5%, 1.90 mM), HZ-HA (46.25%, 1.85 mM), and LQ-HA (43.25%, 1.73 mM), with the above three cultures showing very similar performances. Importantly, the HA-promoted microbial reduction of Fe(III) started with a lag phase of 4–48 h, during which the concentration of Fe(II) increased very slowly, followed by a rapid concentration increase after 48 h and culminating in a plateau after 112–156 h. The above trend was similar to that observed for the biotic control, whereas there is no production of Fe(II) in the abiotic control. The results of dissimilatory Fe(III) reduction of 4 mM FeOOH by MR-1 in the presence of standard HA were shown in Supplementary Figure S2 of Supplementary Material.

## Discussion

Herein, we proved that HAs obtained from four different sediments (LQ-HA, XX-HA, QZ-HA, and HZ-HA) could stably enhance the microbial reduction of nitrate and amorphous FeOOH, with the best performance observed for QZ-HA. The results of elemental analysis, CV, FTIR spectroscopy, and ESR characterization of HAs were in agreement with each other.

Humic acids isolated from sediments of different origins exhibited variable yields and elemental compositions, in agreement with the fact that HSs of different origin show heterogeneity in their compositional and structural characteristics (Thomsen et al., 2002). The properties of HAs in sediments depend on microbial activity, sediment texture, environment productivity, and the extent of bioturbation and accumulation influence on surface sediments (Aiken et al., 1985). Considering atomic ratios, the data in **Table 1** show that the C/N, H/C, and O/C ratio differs widely for different HAs. The C/N, H/C, and O/C are often used to identify HSs from different sources and to follow their structure changes in different environment (Steelink, 1985; He et al., 2008). The C/N ratio depends on the sources of HSs in natural systems, e.g., nonvascular aquatic plants exhibit high C/N ratios, typically between 2.0 and 10.0, whereas vascular terrestrial plants feature C/N ratios of 20 and higher (He et al., 2008), the C/N ratios in **Table 1** ranged from 12.88 to 16.16, indicating that these HSs originated primarily from aquatic plants. The determined C/N values agreed with literature data (7.9–16.2) for sedimentary HAs (Filip et al., 1988), equaling 16.16 for QZ-HA and 12.88 for XX-HA. High C/N ratios also suggest a high degree of condensation, high stability, and an extended degree of organic matter humification (Lu et al., 2000), additionally indicating the predominance of polysaccharides (e.g., lignin) humification (Anuradha et al., 2011). QZ-HA, featuring the highest C/N ratio, also showed the highest nitrate and FeOOH reduction extents. The low C contents of four HAs in this study compared to those literatures (Steelink, 1985; Tatzber et al., 2007) might be attributable to the texture of the sediments with high organometallic compounds or other unknown contents as suggested by the obtained results in **Table 1** (high ash contents contained in HAs) and in **Table 2** (high Fe contents).

The value of H/C ratio can also be considered as a source indicator of organic matter. The H/C values in all HAs are greater than one, which supports the findings of previous studies on HAs (Guo et al., 2018), reflecting that these HSs probably originate from algal or organic matter rather than vascular plant material (Bourbonniere and Meyers, 1983). The O/C ratio is considered as an indicator of oxygen-containing functional group (e.g., carboxylic acid) of HSs, high O/C ratio indicates high carboxyl-C and carbonyl-C composition (Lu et al., 2000). QZ-HA, featuring the highest O/C ratio, may contain the highest oxygen-containing functional group, which contributed to the highest nitrate and FeOOH reduction extents.

The metal content analysis of HAs showed that the level of Fe was positively correlated with extents of DNRA and amorphous FeOOH reduction. QZ-HA showed the best performance in these reactions, which indicated that the inherent redox functionalities of HAs from Qi Zhen lake sediment may be more active and can more effectively facilitate the electron-mediating process for microbial reduction. LQ-HA featured the lowest reduction extent and contained the lowest amount of Fe (9.82 g kg^-1^ sample). Previously, an iron-rich HS was shown to exhibit a stable electron-mediating ability for the microbial reductive dechlorination of pentachlorophenol under anaerobic conditions (Zhang and Katayama, 2012), and a complex synthetized from HA and FeSO_4_ was also shown to exhibit stable redox activity (Zhang et al., 2014). Moreover, immobilized Fe–HA complexes were also demonstrated to act as RMs for iopromide removal from anaerobic sludge (Cruz-Zavala et al., 2016). Therefore, the high Fe content of QZ-HA might contribute to its performance as a RM.

Cyclic voltammetry analyses (**Figure 1**) showed that all HA samples possessed redox-active moieties and exhibited redox potentials between -0.36 and -0.28 V vs. SHE, demonstrating that these moieties belong to the same group despite their heterogeneous properties. The response of quinone moieties on Pt electrodes is known to be improved in DMSO due to its strong chemisorption on Pt (Nurmi and Tratnyek, 2002). Therefore, the improved CV signals of HAs in DMSO were attributed to the presence of quinone-like structures, which have been extensively documented as redox-active moieties (Ratasuk and Nanny, 2007; Uchimiya and Stone, 2009).

Fourier transform infrared analysis revealed that all investigated HAs exhibited similar spectra with different absorbance intensities, indicating that they possessed similar structures and contained the same classes of functional groups regardless of their origin (**Figure 2**). Notably, among all samples, the pronounced band around 1650 cm^-1^, which was ascribed to C=C stretches of aromatic rings, C=O stretches of amide groups (amide I band) and C=O of quinone ketones, and asymmetrical C–O stretches of carboxyl groups, the bands from 870 to 700 cm^-1^ could be attributed to aromatic C–H. These groups are commonly observed for HAs (Senesi et al., 2003; Hesse et al., 2005), they have been reported to be redox-active and might be involved in the action of HAs as RMs (Zhang et al., 2014; Guo et al., 2018). The increase in ESR signals at different pH suggested the presence of semiquinone-type radicals (Scott et al., 1998; Paul et al., 2006; Roden et al., 2010).

*Shewanella oneidensis* is a model dissimilatory iron-reducing bacterium which plays an important role in the biogeochemical cycling of elements and bioremediation because of its diverse respiratory capabilities. The results in **Figures 4, 5** together with Supplementary Figures S1, S2 in the Supplementary Material provide the evidence that dissimilatory nitrate reduction and iron reduction in a pure culture of *S. oneidensis* strain MR1 were enhanced by HAs. During the DNRA process enhanced by HAs, ammonium which is more bio-available was produced, and nitrite accumulation was not observed, this may be beneficial for reducing nitrate leaching and N_2_O emissions in aquatic ecosystems (Rütting et al., 2011). Therefore, HAs in sediments enhanced the recycling of nitrogen used to form ammonium, which also represents *in situ* remediation ability in aquatic ecosystems through microbial redox reactions (Guo et al., 2018). Humic RMs were also reported for the enhancement of denitrification (Xu et al., 2015; Xiao et al., 2016), further study should also be carried out to investigate the effects of HSs as an RM on other microbial metabolic routes in the nitrogen cycle (i.e., nitrogen fixation and ammoniation, which will be helpful for further understanding on the influence of HSs to the total nitrogen cycle in aquatic ecosystems through microbial metabolisms).

Dissimilatory reduction of FeOOH, one of the most predominant terminal electron acceptors of extracellular respiration, plays an important role in the iron biogeochemical processes (Lovley, 1993), and influences the fate of various inorganic and organic environmental pollutants (Li et al., 2010; Zhou et al., 2016). Fe(III) was used as an electron acceptor during dissimilatory iron reduction by iron-reducing bacterium, the produced Fe(II) could couple the oxidation of organic pollutants (i.e., DDT) (Li et al., 2010) and heavy metals [i.e., Mn(IV), Cr(VI), and Hg(II)] (Richter et al., 2012), then organic pollutants were degraded and heavy metals were reduced to a lower valence state or co-precipitated with Fe(II). Notably, the presence of HAs greatly accelerate degradation of organic pollutants and the detoxification of toxic metals, thereby reducing their toxicity. HAs were suggested to be versatile RM, with quinone considered as main redox active groups, which could donate electrons to iron-reducing bacterium utilizing different terminal electron acceptors in microbial respiration (Wolf et al., 2009; Zhang et al., 2014; Wang et al., 2017; Guo et al., 2018).

The above characterization of HAs in this study suggested that the quinone structure in the organic fraction of HAs functioned as RM for the reduction of nitrate and FeOOH, this is consistent with the previous studies (Roden et al., 2010; Zhang C. et al., 2015; Wang et al., 2017). Contributions of other types of electron-mediating moieties such as metal-organic complexes (i.e., ferriporphyrin) could not be excluded, considering the large proportion of Fe. Further study is required to explore the electron-mediating nature of HAs.

## Conclusion

All HAs extracted from different sediments were shown to enhance the microbial reduction of nitrate and FeOOH by *S. oneidensis* MR-1, with both reactions best promoted by QZ-HA, characterized by high C/N, O/C, and Fe ratios. CV analyses confirmed the presence of redox-active moieties in all HAs, and FTIR spectra suggested the aromatic C=C, C–H, and C=O of quinone ketones group in HAs, while ESR spectra verified that quinone moieties within HAs are the redox-active centers. Although the proportion of redox-active carbon is very small, the potential electron-mediating ability is not negligible. This study provided deeper understanding on the role of HAs participating in various biogeochemical processes (i.e., iron cycle, nitrogen cycle, etc.), which also hold great promise for the *in situ* bioremediation.

## Author Contributions

C-FZ and D-DZ conceived and designed the study, and critically revised the manuscript. NZ performed the experiments, analyzed the data, and drafted the manuscript, H-DJ, X-WY, Z-CZ, and S-MY contributed reagents/materials/instruments, study implementation, and manuscript revision. All authors read and approved the final manuscript.

## Conflict of Interest Statement

The authors declare that the research was conducted in the absence of any commercial or financial relationships that could be construed as a potential conflict of interest.
