# Supplementary material for: Redox Structures of Humic Acids Derived From Different Sediments and Their Effects on Microbial Reduction Reactions
Source: Front Microbiol. 2018 Jun 8;9:1225. doi: 10.3389/fmicb.2018.01225 (PMC6002622; doi:10.3389/fmicb.2018.01225)
Supplement: Supplementary file 1 [file Data_Sheet_1.docx]

Supplementary Material

Redox structures of humic acids derived from different sediments and their effects on microbial reduction reactions

Ning Zhang *, Dong-Dong Zhang*, Hong-Da Ji, Xin-Wei Yu, Zhi-Chao Zhang, Sheng-Mao Yang

*** Correspondence:** Chun-Fang Zhang: zhangcf@zju.edu.cn

**Table S1 Physicochemical characteristics of sampling sites and interstitial water in the sediments**

| Sampling Site | Latitude  （N） | Longitude  （E） | Depth（m） | E  (mv） | Temperature（°C） | Salinity  (‰) | pH | DO（mg/L） | TDS（mg/L） | Conductivity（μs/cm） | Resistvity（KΩ·cm） |
| --- | --- | --- | --- | --- | --- | --- | --- | --- | --- | --- | --- |
| Ling Qiao river | 29° 51′59″ | 121°33′35″ | 2m | -6.00 | 18.50 | 0.33 | 7.76 | 0.14 | 325.00 | 667.00 | 1.50 |
| Xi Xi wetland | 30° 1527″ | 120° 3′ 25″ | 1m | -6.10 | 19.20 | 0.21 | 7.48 | 0.07 | 211.80 | 439.00 | 2.27 |
| Qi Zhen lake | 30° 18′ 28″ | 120° 5′ 3″ | 1.5m | -2.80 | 18.30 | 0.17 | 7.40 | 0.16 | 172.10 | 361.00 | 2.82 |
| Hu Zhou pond | 30° 46′ 38″ | 120° 9′ 5″ | 1.8m | -6.20 | 19.40 | 0.58 | 7.14 | 0.05 | 576.00 | 1219.00 | 0.83 |

**Fig. S1|** the changes in the concentration of nitrate (**A**), nitrite (**B**), ammonium (**C**) left in the culture within 72 h, in which microbial reduction were conducted by MR-1 in the presence of standard HA (Aldrich) when 5mM nitrate was added as the electron acceptor in each culture. Data show the mean values of triplicate cultures, and vertical bars show the difference in triplicate cultures.

**Fig. S2|** Production of total Fe (II) during the microbial reduction of FeOOH conducted by MR-1 in the presence of standard HA (Aldrich) within 156 hours. Data show the mean values of triplicate cultures, and vertical bars show the difference in triplicate cultures.


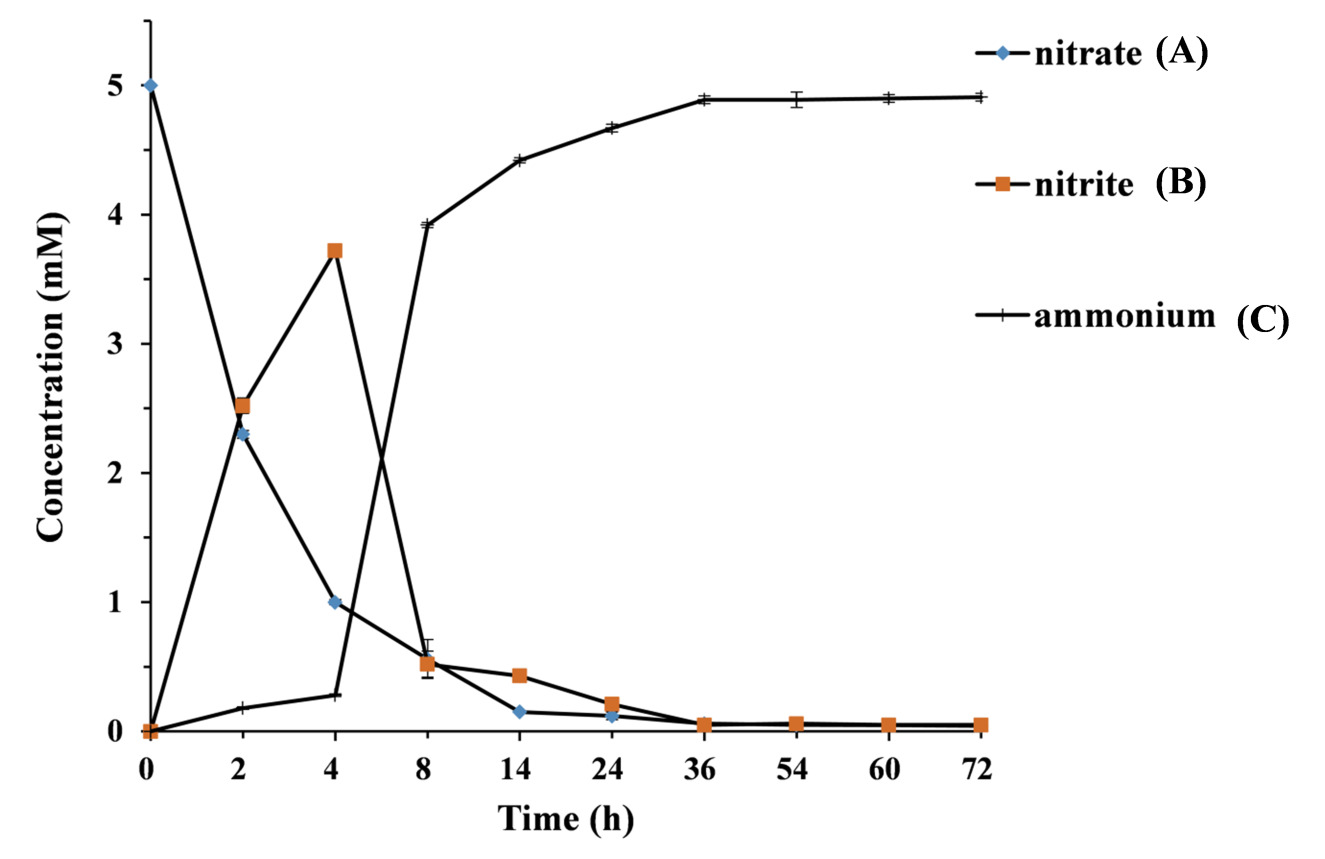


**Fig. S1**


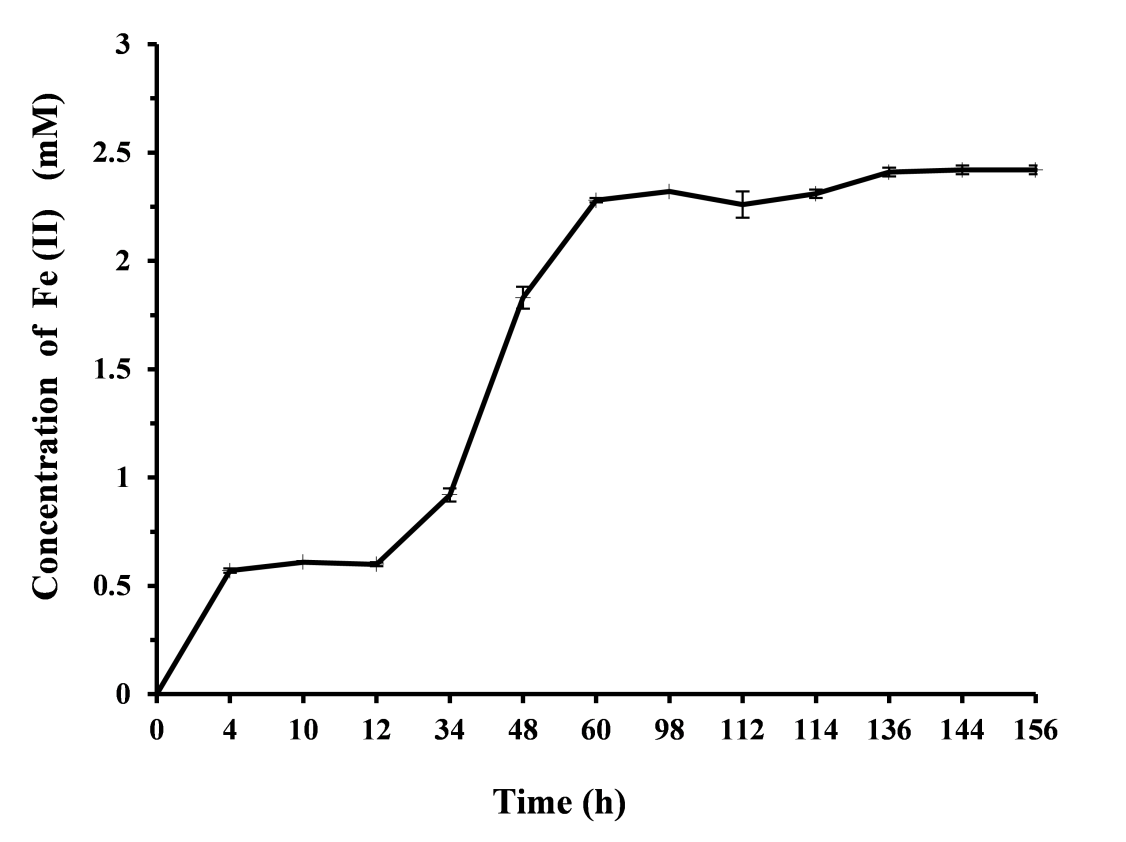


**Fig. S2**
